# Prevalence of rhesus D-negative blood type and the challenges of rhesus D immunoprophylaxis among obstetric population in Ethiopia: a systematic review and meta-analysis

**DOI:** 10.1186/s40748-021-00129-3

**Published:** 2021-02-02

**Authors:** Asteray Assmie Ayenew

**Affiliations:** Midwifery Department, College of Medicine and Health Sciences, Bahir Dar University, Bahir Dar, Ethiopia

**Keywords:** Rhesus isoimmunization, Rh-negative women, Universal access, Anti-D, Management, Ethiopia

## Abstract

**Background:**

Transplacental or fetomaternal hemorrhage (FMH) may occur during pregnancy or at delivery and lead to immunization to the D antigen if the mother is Rh-negative and the baby is Rh-positive. This can result in hemolytic disease of the fetus and newborn (HDFN) in subsequent D-positive pregnancies. Therefore, the aim of this systematic review and meta-analysis was to estimate distribution of ABO and Rh (D) blood groups among pregnant women in Ethiopia.

**Method:**

We searched PubMed, Google Scholar, EMBASE, Cochrane Library, HINARI, AFRO Library Databases, and African Online Journal databases for all available studies using the following keywords: “High rhesus (Rh(D)) negative frequency”, “ABO blood group distribution”, “haemolytic disease of the newborn (HDN)”, “rh isoimmunization”, “anti-RhD immunoglobulin”, “D-negative pregnancies”, “Frequency”, “ABO and Rh blood group distribution”, “feto-maternal hemorrhage”, “rhesus D negative pregnant mothers”, “kleihauer-betke test (KBT)”, “Neonatal Hyperbilirubinemia”, “non-sensitized RhD-negative pregnant women”, “antenatal anti-D immunoglobulin prophylaxis”, “Hemolytic disease of the newborn (alloimmunization), Ethiopia. The search string was developed using “AND” and “OR” Boolean operators. All published and unpublished observational studies reporting the distribution of ABO and Rh (D) blood groups among pregnant women in Ethiopia were included. The study participants were all pregnant women in Ethiopia, and the main outcome measure of this systematic review and meta-analysis was Rhesus D-negative blood type and ABO blood group distribution among pregnant women in Ethiopia. The data was extracted by the author (AAA) by using a standardized JBI data extraction format. Microsoft Excel (2016), and Stata version 11.0 (Stata Corporation, College Station, Texas, USA) software were used for data entry and analysis, respectively. The random effect model was used for estimating the pooled effects, and the publication bias was assessed by visual inspection of the funnel plots and objectively by using the Egger’s test (i.e. *p* < 0.05).

**Results:**

One hundred thirty-two articles were identified through electronic database searching. Of which, 34 were excluded due to duplication, 65 through review of titles and abstracts, and 22 full-text articles were excluded for not reporting the outcome variable and other reasons. Finally, 7 were included to estimate the distribution of ABO and Rh (D) blood groups among pregnant women in Ethiopia. The pooled distribution of Rh-negative blood group among pregnant women in Ethiopia was 10.8% (95%CI: 7.53**–**14.07, I^2^ = 85%, *p* < 0.001). In the ABO system, type O was the most prevalent 39.9% (37.51**–**42.38), followed by A (30.59% (26.00**–**35.18)), B (23.04% (20.03**–**26.05)), and AB the least (4.82%(3.17**–**6.47)), in the pattern O > A > B > AB.

**Conclusion:**

The pooled distribution of Rh-negative blood group among pregnant women in Ethiopia was high. Rh alloimmunization remains a major factor responsible for perinatal morbidity in Ethiopia and may result in the compromise of the woman’s obstetric care due to the unaffordability of anti-D immunoglobulin. There is the urgent need for the implementation of universal access to anti-D immunoglobulin for the Rh-negative pregnant population in Ethiopia.

## Introduction

Isoimmunization is the process of immunizing an individual with antigen derived from the similar subject, provided that the said antigen was initially absent. The Rhesus (Rh) antigen is found on the surface of human red blood cell (RBC) membrane [1, 2]. The ABO system and the Rhesus (Rh) system remain the most clinically significant blood group antigens on the red cell membrane. If the mother is RhD-negative and the fetus RhD positive, she has a potential capacity to form antibodies if exposed to fetal antigens, a process known as RhD sensitization [2–5].

Alloimmune hemolytic disease of the fetus and newborns (HDF/N) results from the destruction of red cells by maternal immunoglobulin (IgG) antibodies that gain access to the fetal circulation during gestation. The most serious form of HDFN is caused by maternal alloantibodies directed against the D antigen of the Rh blood group system due to the high immunogenicity of D antigen [3–5].

In Ethiopia, there is poor and sometimes no alloimmunization prevention following potentially sensitizing events, and during medical termination of pregnancy in Rh-negative women. Information about previous pregnancies and termination of pregnancy are often lacking in patients’ medical notes due to poor data management. These issues have made the management of Rh-negative pregnancy a huge challenge [6]. Despite the fact that the prevalence of Rh-negative phenotype is significantly lower among Africans than Caucasians, the Rh alloimmunization remains a major factor responsible for perinatal morbidity in Ethiopia, and resulted in the compromise of the woman’s obstetric care due to the unaffordability of anti-D immunoglobulin [7]. Even though, the risk of sensitization depends largely upon the extent of the maternal immune response, volume of transplacental haemorrhage, and concurrent presence of ABO incompatibility [8], there is the urgent need for the implementation of universal access to anti-D immunoglobulin for the Rh-negative pregnant population in Ethiopia [9, 10]. Anti-D immunoglobulin should be available in cases of potentially sensitizing events such as amniocentesis, cordocentesis, antepartum hemorrhage, vaginal bleeding during pregnancy, external cephalic version, abdominal trauma, intrauterine death and stillbirth, inutero therapeutic interventions, miscarriage, and therapeutic termination of pregnancy [11, 12]. There is also the need for the availability of Feto-Maternal Hemorrhage measurements following potentially sensitizing events. The low-cost acid elution method, a modification of the Kleihauer–Betke (KB) test, can become a readily available, affordable, and minimum alternative to flow cytometric measurement of FMH. Knowledge of anti-D prophylaxis among obstetricians, biomedical scientists, midwives, traditional birth attendants, pharmacists, and nurses in Ethiopia needs to be improved. This will facilitate quality antenatal and postnatal care offered to Rh-negative pregnant population and improve perinatal outcomes [13].

To prevent sensitization, all D-negative women who deliver a D-positive fetus should receive at least a single 300-μg dose of RhIG within 72 h of delivery. In addition, a maternal sample should be obtained approximately 1 h after delivery and tested for evidence of a FMH in excess of 30 mL of fetal blood. Approximately 17% of Rh D–negative women who deliver Rh D–positive fetus become alloimmunized if RhIG is not administered appropriately. RhIG prophylaxis has reduced the overall risk of Rh immunization from 13.2 to 0.2%, and testing for large FMH has further decreased the risk to 0.14%. Hence, RhD immunization may be further reduced by strict compliance to guidelines concerning determination of FMH and accordingly adjusted RhIG or by routine administration of extra RhIG after a non-spontaneous delivery and/or a complicated or prolonged third stage of labor [3, 5, 14].

It is part of modern antenatal care to give all RhD-negative pregnant women an anti-RhD immunoglobulin IgG injection at about 28 week’s gestations with a booster at 34 weeks gestation [2]. However, In Ethiopia this could not given because of unaffordablity of anti-RhD immunoglobulin IgG. Thus, Rh alloimmunization remains a major factor responsible for perinatal morbidity, compromise of the woman’s obstetric care due to the unaffordability of RhIG, and divorce in Ethiopia. Therefore the aim of this systematic review and meta-analysis was to estimate the prevalence of Rhesus D-negative blood type among obstetric population in Ethiopia.

## Methods

This systematic review and meta-analysis were conducted to estimate the distribution of ABO and Rh-Negative blood groups among pregnant women in Ethiopia. We used the Preferred Reporting Items for Systematic Reviews and Meta-Analyses (PRISMA) checklist guideline [15].

### Searching strategy

First, the PROSPERO database and database of abstracts of reviews of effects (DARE) (http://www.library.UCSF.edu) were searched to check whether published or ongoing projects exist related to the topic. The literature search strategy, selection of studies, data extraction, and result reporting were done in accordance with the Preferred Reporting Items for Systematic Reviews and Meta-Analyses (PRISMA) guidelines [16]. We searched PubMed, Google Scholar, EMBASE, Cochrane Library, HINARI, AFRO Library Databases, and African Online Journal databases for all available studies using the following keywords: “High rhesus (Rh(D)) negative frequency”, “ABO blood group distribution”, “haemolytic disease of the newborn (HDN)”, “rh isoimmunization”, “anti-RhD immunoglobulin”, “management”, “D-negative pregnancies”, “Frequency”, “ABO and Rh blood group distribution”, “feto-maternal hemorrhage”, “rhesus D negative pregnant mothers”, “kleihauer-betke test (KBT)”, “Neonatal Hyperbilirubinemia”, “non-sensitized RhD-negative pregnant women”, “flow cytometry (FCM)”, “antenatal anti-D immunoglobulin prophylaxis”, “Hemolytic disease of the newborn (alloimmunization), Ethiopia. The search string was developed using “AND” and “OR” Boolean operators. Searching terms were based on adapted PICO principles to search through the above-listed databases to access the relevant articles. For unpublished studies, the official website of Ethiopian’s University research repository online library (University of Gondar and Addis Ababa University) were used.

### Eligibility criteria

#### Inclusion criteria

Study Design: All observational studies reporting the distribution of ABO and Rh (D) blood groups among pregnant women were included.

Language: English language literature and research articles were included.

Publication: Both unpublished and published research articles were considered.

Searching date: Articles searched from June 1–30, 2020 were included.

Study Participants: pregnant women in Ethiopia.

#### Exclusion criteria

Duplicated studies, articles without full text and abstract, anonymous reports, qualitative studies, and case reports were excluded.

### Quality assessment

After collecting the findings from all databases, the articles were exported to Microsoft Excel spreadsheet. The methodological quality of each study (sampling strategy, response rate, and representativeness of the study), comparability, and outcome were checked using the NOS tool. Newcastle-Ottawa Quality Assessment Scale (NOS) for cross-sectional studies was used to assess the methodological quality of a study and to determine the extent to which a study has addressed the possibility of bias in its design, conduct and analysis [17]. All the included articles scored (NOS) 7 and more can be considered as “good” studies with low risk.

### Data extraction

Microsoft Excel (2016), and Stata version 11.0 (Stata Corporation, College Station, Texas, USA) software were used for data entry and analysis, respectively. The data was extracted by the author (AAA) by using a standardized JBI data extraction format. During data extraction; name of the author, sample size, publication year, study design, prevalence, response rate, population outcome, study site, and different contributing factors were included. Moreover, distribution of ABO and Rh (D) blood groups among pregnant women with 95%CI were collected [18].

### Statistical analysis

As the test statistic showed high heterogeneity among studies (I^2^ = 85.0%, *p* < 0.05) the Random-effects model was used to estimate the DerSimonian and Laird’s pooled effect [19]. Cochran’s Q chi-square statistics and I^2^ statistical test was conducted to assess the random variations between primary studies [20]. In this study, heterogeneity was interpreted as an I^2^ value of 25% = low, 50% = moderate, and 75% = high [21]. Potential publication bias was assessed by visually inspecting funnel plots and objectively using the Egger’s test (i.e. *p* < 0.05) [22]. To account for any publication bias, we used the trim-and-fill method, based on the assumption that the effect sizes of all the studies are normally distributed around the center of a funnel plot. The meta-analysis was performed using the Stata version 11.0 (Stata Corporation, College Station, Texas, USA) software. Finally, for all analyses, *P* < 0.05 was considered statistically significant.

## Results

### Study selection and data extraction

**Fig. 1:**
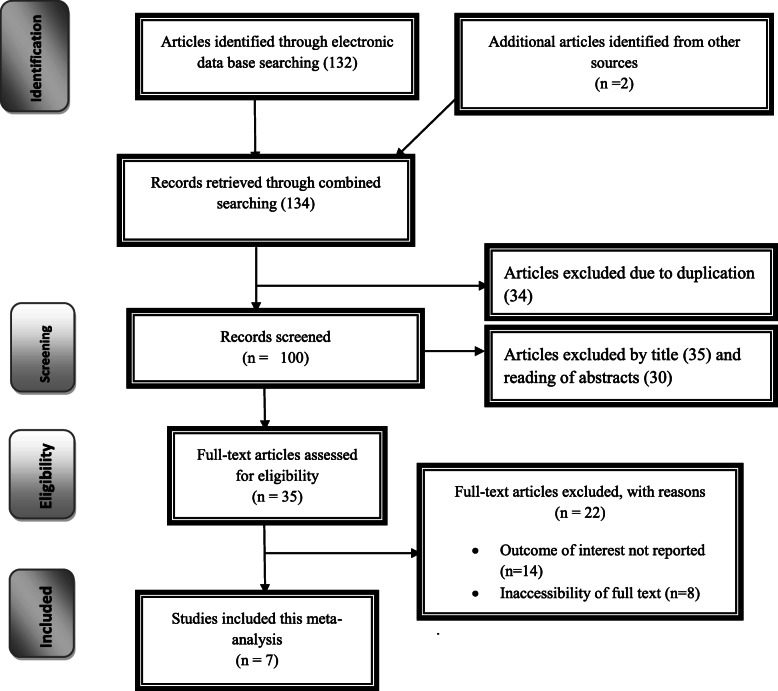
Flow chart of study selection for systematic review and meta-analysis of the distribution of ABO and Rh-Negative blood groups among pregnant women in Ethiopia

The search strategy identified 56 articles from PubMed, 43 articles from Google Scholar, 25 articles from Cochrane Library, 10 articles from African Journals Online, and 5 articles from Ethiopian’s University online library. Of which, 34 were excluded due to duplication, 65 through review of titles and abstracts. Additionally, 22 full-text articles were excluded for not reporting the outcome variable and other reasons. Finally, 7 were included to estimate the distribution of ABO and Rh (D) blood groups among pregnant women in Ethiopia [Fig. 1].

### Study characteristics

In this review, 7 relevant studies were included with a sample size of 7, 885. All included studies were cross-sectional in study design. Regarding the geographical area; three from Addis Ababa, two from Southern Nation Nationalities and People (SNNPR), and one from Tigray region, one study from Gambella region. Among the included studies, the largest sample size was 5, 987, where as the smallest was 86 (Table 1).

**Table 1:** Descriptive summary of the seven included studies in the systematic review and meta-analysis

Authors	Study area	Study design	Sample size	Response rate	Prevalence (%)	NOS quality score
Lemu G.et al. [10]	Gambella	Hospital based cross sectional study design	449	98.7	19.37	9
Tsegaw, B. et al. [23]	Addis Ababa	Hospital based cross sectional study design	497	87.8	7.2	8
Kebreab P.et al. [24]	SNNPR	Hospital based retrospective descriptive study design	270	100	7	9
Tesfaye K. et al. [25]	SNNPR	community- based cross-sectional study design	441	94.3	8.61	9
Fekadu U. et al. [7]	Addis Ababa	Hospital- based cross-sectional study design	86	97	8.7	9
Kedir M. et al. [26]	Addis Ababa	Hospital based retrospective descriptive study design	155	100	10.3	9
Mgbaru A. et al. [9]	Tigray	Hospital- based cross-sectional study design	5897	91.2	8.8	9

### Distribution of Rh-negative blood groups among pregnant women in Ethiopia

**Fig. 2:**
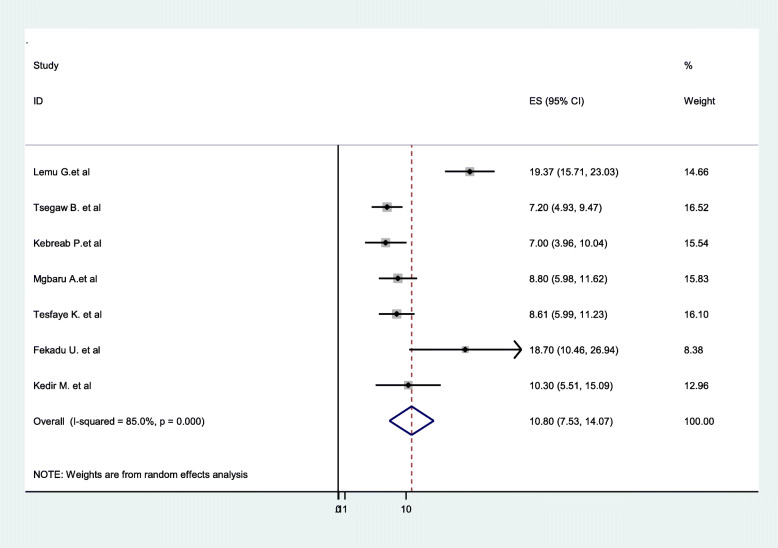
Forest Plot for the pooled distribution of Rh-negative blood groups among pregnant women in Ethiopia, 2020

The pooled distribution of Rh-Negative blood groups among pregnant women is presented on a forest plot (Fig. 2). Therefore, the estimated distribution of Rh-Negative blood group among pregnant women in Ethiopia was 10.8% (95%CI: 7.53**–**14.07, I^2^ = 85%, *p* < 0.001).

### Publication bias

**Fig. 3:**
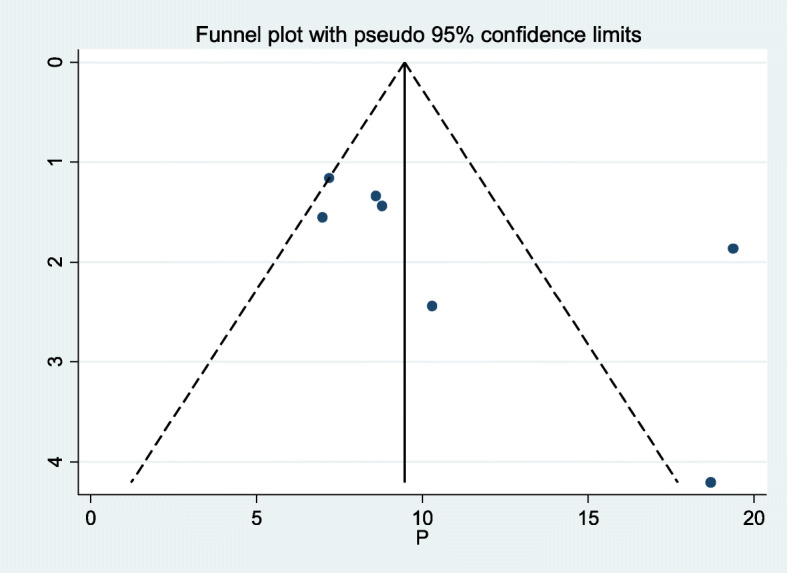
Funnel plot with 95% confidence limits of the pooled distribution of Rh-negative blood groups among pregnant women in Ethiopia, 2020

The funnel plot was assessed for asymmetry distribution of Rh-negative blood groups among pregnant women in Ethiopia (Fig. 3). Egger’s regression test showed a *p*-value of 0.759 with no evidence of publication bias.

### Sensitivity analysis

**Fig. 4:**
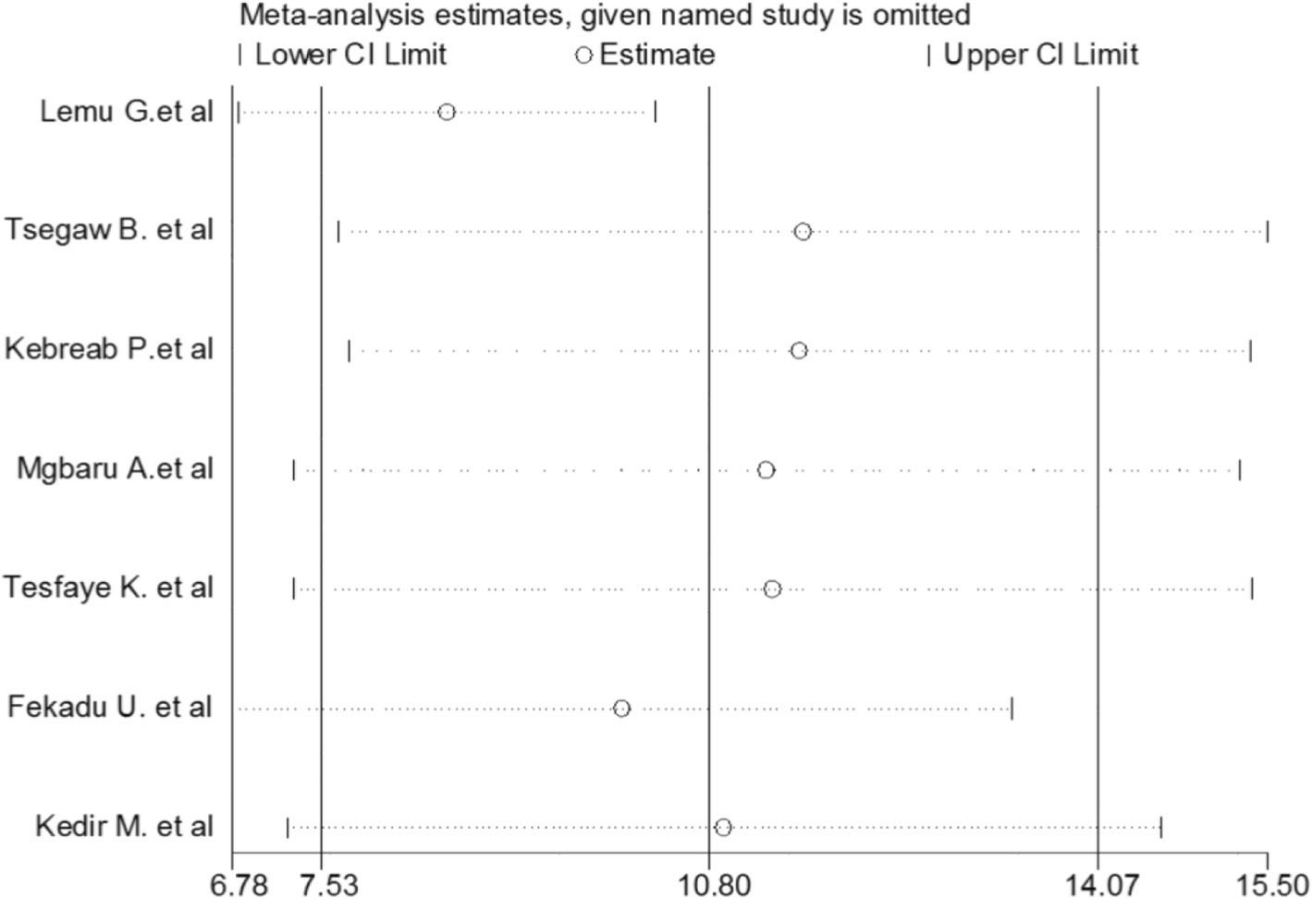
Sensitivity analysis of the pooled distribution of Rh-negative blood groups among pregnant women in Ethiopia, 2020

This systematic review and meta-analysis showed that the point estimate of its omitted analysis lies within the confidence interval of the combined analysis. Therefore, trim and fill analysis was no further computed (Fig. 4).

### Distribution of ABO blood groups among pregnant women

**Fig. 5:**
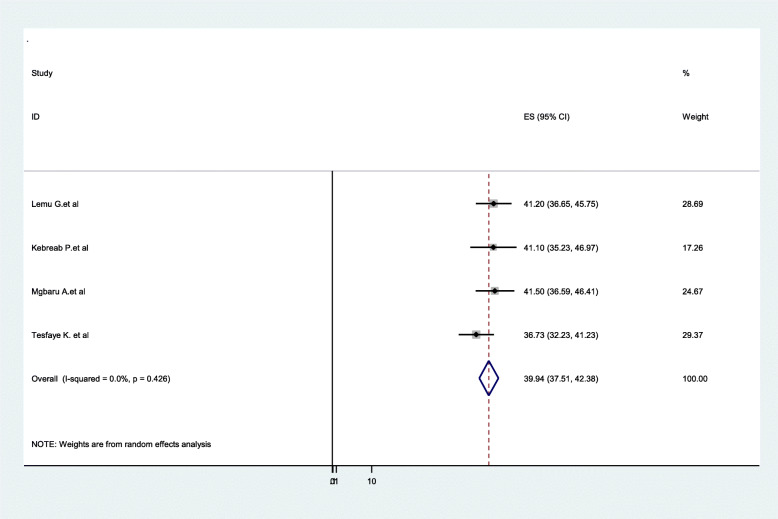
Forest Plot for the pooled distribution of O blood group among pregnant women in Ethiopia, 2020

**Fig. 6:**
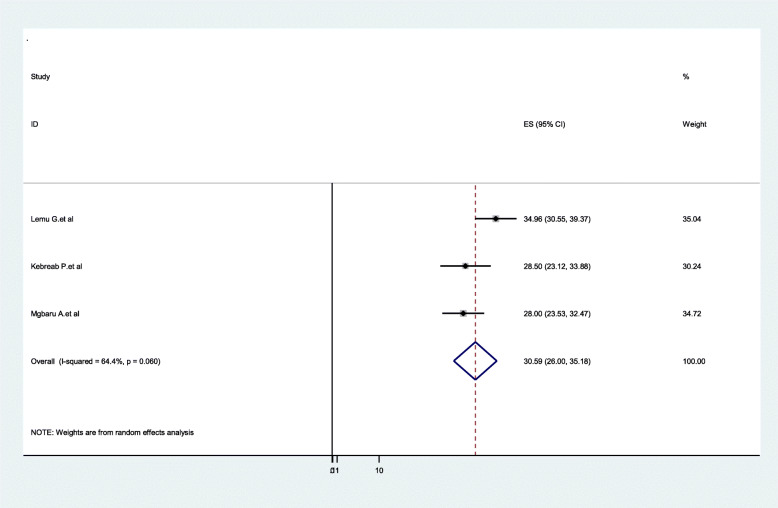
Forest Plot for the pooled distribution of A blood group among pregnant women in Ethiopia, 2020

In the ABO system, type O was the most prevalent 39.9% (95%CI:37.51**–**42.38, I^2^ = 0.00%, *P* = 0.426). A total of four articles were included to pool the distribution of O blood group among pregnant women in Ethiopia. Moreover, a total of three articles were included to pool the distribution of A blood group among pregnant women, and the distribution was 30.59% (95%CI:26.00**–**35.18, I^2^ = 64.4%, *P* = 0.06) (Figs. 5 & 6).

**Fig. 7:**
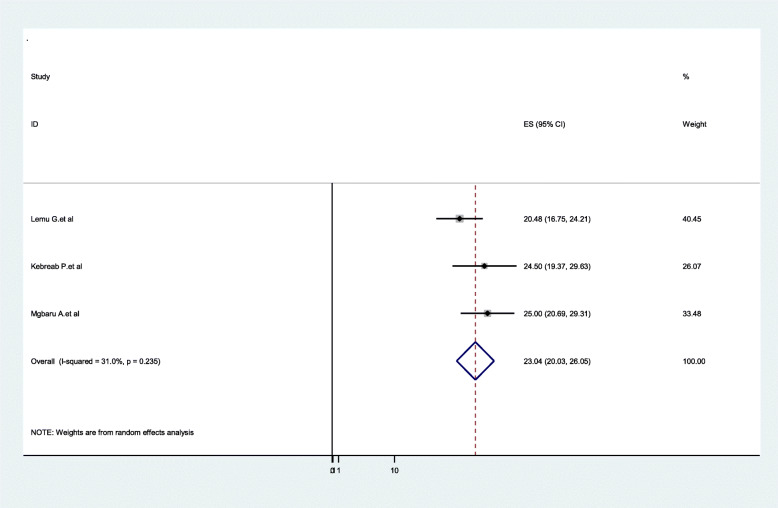
Forest Plot for the pooled distribution of B blood group among pregnant women in Ethiopia, 2020

**Fig. 8:**
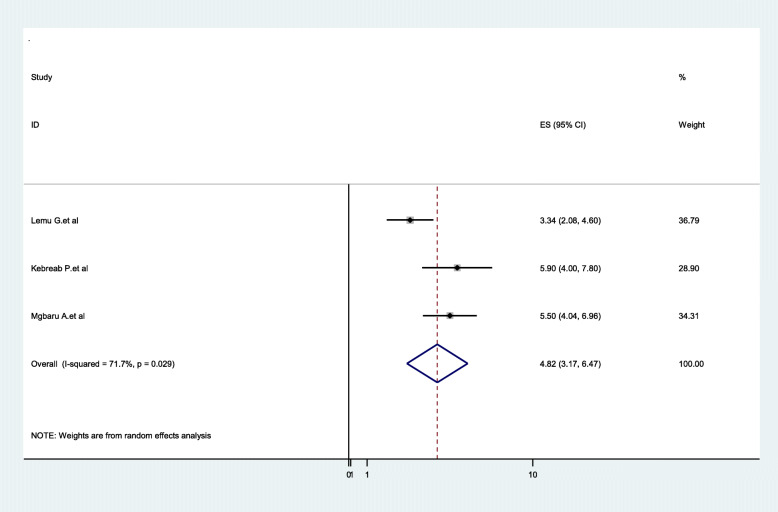
Forest Plot for the pooled distribution of AB blood group among pregnant women in Ethiopia, 2020

Three studies showed the pool the distribution of B blood group among pregnant women in Ethiopia, with the overall distribution of 23.04% (95%CI:20.03**–**26.05, I^2^ = 31.0%, *P* = 0.235)), and three were also included for the AB blood group distribution with the pooled prevalence of 4.82% (95%CI:3.17**–**6.47, I^2^ = 71.7%, *P* = 0.029) (Figs. 7 & 8).

## Discussion

RhD-positive red blood cells (containing the D antigen) from the fetus stimulate antibody production in RhD-negative mothers. During pregnancy and delivery of the first RhD-positive fetus to RhD-negative mothers, the red blood cells of the RhD-positive fetuses enter the RhD-negative mothers and stimulate the mothers to produce IgG anti-D antibodies. When the RhD-negative mother later carries an RhD-positive fetus, the antibodies in the maternal serum enter the fetal blood circulation via the placental barrier, and can cause neonatal hemolysis [27].

Hemolytic disease of the fetus and newborn (HDFN) can lead to fetal hemolytic anemia, jaundice, intellectual retardation, premature birth, abortion, and stillbirth. HDFN is an important cause of neonatal morbidity and death [28–30]. To reduce the incidence of HDFN and mortality among fetuses and neonates, anti-D immunoglobulin has been tested in clinical trials 1960s. Anti-D immunoglobulin has been used to prevent postpartum disease in RhD-negative women, and has greatly reduced HDFN-related morbidity as well as fetal and neonatal mortality [31].

However, during pregnancy with the first RhD-positive fetus, or within 72 h after delivery, RhD-negative mothers can be intramuscularly injected with 300 μg anti-D immunoglobulin, which can bind to the D antigen leaked into the mother’s serum and desensitize it, thus blocking anti-D antibody production in the mother’s serum. Anti-D immunoglobulin had no significant preventive effect on mothers who had already produced anti-D antibodies [32].

In Ethiopia, antenatal care coverage is low, home childbirth is very high, knowledge of Rh isoimmunization is poor, and unaffordablity of anti-D immunoglobulin results many complication on the newborn, mothers, and families. Therefore, the aim of this systematic review and meta-analysis was to estimate the prevalence of Rhesus D-negative blood type among the obstetric population in Ethiopia. Thus, the pooled distribution of Rh-negative blood group among pregnant women in Ethiopia was 10.8%. The result lower is than the studies conducted in Western nations like Britain [33] and United States [34] have the Rh factor negativity of 17 and 15% respectively.

On the other hand the result of this study is higher than the studies conducted in France [35] 0.9%, Nigeria [36] 4.44%, Kenya [37], Guinea [38] 4.06, 2.4% in Cameroon [39]. Despite the high prevalence of Rh negative blood group among pregnant women in Ethiopia, receiving anti-D immunoglobulin immunoprophylaxis is very low due to low antenatal care service, high rate of home delivery, and financial constraints. The other reasons given for not receiving immunoprophylaxis, apart from financial inability, showed the poor knowledge of the women about Rhesus isoimmunization, and there is need to improve their knowledge via the antenatal health counseling [6].

Moreover, blood grouping and cross matching of the ABO blood group should be performed in all pregnant women at the first visit. If the woman is Rh-D positive no further testing for blood groups is required. In western countries testing for antibodies against all the Rh red cell antigens (D, Ee, Cc, Kell, Duffy, Kidd, Jka, Jkb and M) is also performed irrespective of the blood group and if a woman is positive for these antibodies she managed as a case of isoimmunized pregnancy. However because of the high cost, this test is not routinely performed in Ethiopia. It is reserved for pregnant women, who are Rh positive or Rh-D negative with negative indirect Coombs test (ICT) for Rh-D antibodies, with a past obstetric history suggestive of isoimmunization (birth of a baby with features of hydrops, neonatal jaundice or history of postnatal exchange transfusion).

## Limitation

Since it is the first systematic review and meta-analysis, it is taken as strength. The included articles were restricted to the English language only; this is a limitation of the study as it missed studies published in local languages.

## Conclusion

The pooled distribution of Rh-negative blood groups among pregnant women in Ethiopia was high. There is an urgent need for the implementation of universal access to anti-D immunoglobulins for the Rh-negative pregnant population in Ethiopia. Moreover, anti-D immunoglobulin should be available in cases of potentially sensitizing events such as amniocentesis, cordocentesis, antepartum hemorrhage, vaginal bleeding during pregnancy, external cephalic version, abdominal trauma, intrauterine death and stillbirth, in utero therapeutic interventions, miscarriage, and therapeutic termination of pregnancy.

## Data Availability

The data sets generated during the current study are available from the corresponding author on reasonable request.

## Abbreviations

AA: Addis Ababa
CI: Confidence Interval
PRISMA: Preferred Reporting Items for Systematic Reviews and Meta-Analyses
RH: Rhesus factor
SNNPR: Southern Nation Nationality and Peoples Representatives

## References

[1] EAea NAGAMUTHU (2017). Prevalence of rhesus negativity among pregnant women. Int J Res Med Sci.

[2] Hitzeroth HW, Op’t Hof J (1988). On the prevention of rhesus immunisation in the RSA. South Afr Med J.

[3] Greer JP, Arber DA, Glader BE, List AF, Means RM, Rodgers GM (2018). Wintrobe’s clinical hematology: Lippincott Williams & Wilkins.

[4] Pourazar A, Homayouni V, Rezaei A, Andalib A, Oreizi F (2008). The assessment of feto-maternal hemorrhage in an artificial model using anti-D and anti-fetal hemoglobin antibody by flow cytometry. Iran Biomed J.

[5] Lafferty JD, Raby A, Crawford L, Linkins LA, Richardson H, Crowther M (2003). Fetal-maternal hemorrhage detection in Ontario. Am J Clin Pathol.

[6] Osaro E, Charles AT (2010). Rh isoimmunization in sub-Saharan Africa indicates need for universal access to anti-RhD immunoglobulin and effective management of D-negative pregnancies. Int J Women's Health.

[7] Urgessa F, Tsegaye A, Gebrehiwot Y, Birhanu A (2014). Assessment of feto-maternal hemorrhage among rhesus D negative pregnant mothers using the kleihauer-betke test (KBT) and flow cytometry (FCM) in Addis Ababa, Ethiopia. BMC Pregnancy Childbirth.

[8] Roman AS (2013). Late pregnancy complications. In: Decherney AH ea. current obstetrics and gynecologic, diagnosis and treatment.

[9] Alemu M, Abrha G, Bugssa G, Tedla K (2014). Frequency of ABO and Rh (D) blood groups and hemoglobin threshold among pregnant women in family guidance association, Mekelle model clinic, North Ethiopia.

[10] Golassa L, Tsegaye A, Erko B, Mamo H (2017). High rhesus (Rh(D)) negative frequency and ethnic-group based ABO blood group distribution in Ethiopia. BMC Res Notes.

[11] Koelewijn JM, de Haas M, Vrijkotte TG, van der Schoot CE, Bonsel GJ (2009). Risk factors for RhD immunisation despite antenatal and postnatal anti-D prophylaxis. BJOG.

[12] Tiblad E, Taune Wikman A, Ajne G, Blanck A, Jansson Y, Karlsson A (2013). Targeted routine antenatal anti-D prophylaxis in the prevention of RhD immunisation--outcome of a new antenatal screening and prevention program. PLoS One.

[13] Cortey A, Brossard Y (2006). Prevention of fetomaternal rhesus-D Allo-immunization. Practical aspects. J de Gynecol.

[14] Judd WJ (2001). Practice guidelines for prenatal and perinatal immunohematology, revisited.

[15] Moher D, Liberati A, Tetzlaff J, Altman D (2009). Preferred reporting items for systematic reviews and meta-analyses: the PRISMA statement. Public Libr Sci Med.

[16] Moher D, Liberati A, Tetzlaff J, Altman DG (2009). Preferred reporting items for systematic reviews and meta-analyses: the PRISMA statement. PLoS Med.

[17] Downes MJ, Brennan ML, Williams HC, Dean RS (2016). Development of a critical appraisal tool to assess the quality of cross-sectional studies (AXIS). BMJ Open.

[18] Sendeku FW, Azeze GG, Fenta SL (2020). Perinatal asphyxia and its associated factors in Ethiopia: a systematic review and meta-analysis. BMC Pediatr.

[19] DerSimonian R, Laird N (1986). Meta-analysis in clinical trials. Control Clin Trials.

[20] Huedo-Medina TB, Sánchez-Meca J, Marín-Martínez F, Botella J (2006). Assessing heterogeneity in meta-analysis: Q statistic or I2 index?. Psychol Methods.

[21] Higgins JPAD (2008). Assessing risk of bias in included studies. Cochrane handbook for systematic reviews of interventions: Cochrane book series.

[22] D-SG EM, Altman D. Systematic reviews in health care: meta-analysis in context: John Wiley & Sons; 2008.

[23] Tsegaw B (2015). Prevalence of rhesus negative gene among pregnant women and assessment of effective management of RH negative pregnancy in Gandhi memorial hospital, Addis Ababa, Ethiopia.

[24] Chanko KP (2020). Frequency of ABO blood group and Rh (D) negative mothers among pregnant women attending at antenatal Care Clinic of Sodo Health Center, SNNPR, Ethiopia. Am J Clin Exp Med.

[25] Tesfaye K, Petros Y, Andargie M (2015). Frequency distribution of ABO and Rh (D) blood group alleles in Silte zone, Ethiopia. Egyptian J Med Hum Genet.

[26] MONJOR KT (2020). Telahun. . The pattern of ABO incompatibility in neonates in Tikur Anbessa specialized hospital (TASH), Addis Ababa. . Ethiopian. J Pediatr Child Health.

[27] Freda VJ, Gorman JG, Pollack W, Robertson JG, Jennings ER, Sullivan JF (1967). Prevention of Rh Isoimmunization: Progress report of the clinical trial in mothers. JAMA..

[28] Bowman J (2003). Thirty-five years of Rh prophylaxis. Transfusion..

[29] Woodrow JC, Donohoe WT (1968). Rh-immunization by pregnancy: results of a survey and their relevance to prophylactic therapy. Br Med J.

[30] Mollison P, Walker W (1952). Controlled trials of the treatment of haemolytic disease of the newborn. Lancet.

[31] Levine P (1959). The protective action of ABO incompatibility on Rh Isoimmunization and Rh hemolytic disease-theoretical and clinical implications. Am J Hum Genet.

[32] Xie XFQ, Bao Z, Zhang Y, Zhou D (2020). Clinical value of different anti-D immunoglobulin strategies for preventing Rh hemolytic disease of the fetus and newborn: a network meta-analysis. PLoS One.

[33] TF F, Blood groups (ABO groups). Common laboratory and diagnostic tests. 3rd ed. Philadelphia: Lippincott. IJCMPH. p. 2002.

[34] Garratty G, Glynn SA, McEntire RABO (2004). Rh(D) phenotype frequencies of different racial/ethnic groups in the United States. Transfusion..

[35] Liumbruno GM, D’Alessandro A, Rea F, Piccinini V, Catalano L, Calizzani G (2010). The role of antenatal immunoprophylaxis in the prevention of maternal-foetal anti-Rh(D) alloimmunisation. Blood Transfus.

[36] Jeremiah ZA (2005). An assessment of the clinical utility of routine antenatal screening of pregnant women at first clinic attendance for haemoglobin genotypes, haematocrit, ABO and Rh blood groups in Port Harcourt, Nigeria. Afr J Reprod Health.

[37] Mwangi J (1999). Blood group distribution in an urban population of patient targeted blood donors. East Afr Med J.

[38] Loua A, Lamah MR, Haba NY, Camara M (2007). Frequency of blood groups ABO and rhesus D in the Guinean population. Transfus Clin Biol.

[39] Tagny CT, Fongué VF, Mbanya D (2009). The erythrocyte phenotype in ABO and Rh blood groups in blood donors and blood recipients in a hospital setting of Cameroon: adapting supply to demand. Rev Med Brux.

